# Protective Adaptive Immunity Against Severe Acute Respiratory Syndrome Coronaviruses 2 (SARS-CoV-2) and Implications for Vaccines

**DOI:** 10.7759/cureus.8399

**Published:** 2020-06-01

**Authors:** Christopher Manners, Erick Larios Bautista, Hannah Sidoti, Osvaldo J Lopez

**Affiliations:** 1 Medical Sciences, Hackensack Meridian School of Medicine at Seton Hall University, Nutley, USA

**Keywords:** covid-19, sars-cov-2, neutralizing antibodies, vaccines, t-cell immunity

## Abstract

Severe acute respiratory syndrome coronavirus 2 (SARS-CoV-2) is an emerging human coronavirus responsible for coronavirus disease 2019 (COVID-19), a predominantly respiratory disease that has become a global pandemic. Millions of people worldwide are suffering from COVID-19, and hundreds of thousands of those infected have died. Nevertheless, many more people who have been infected with SARS-CoV-2 are asymptomatic or suffer a mild disease characterized by dry cough and mild fever. This new pandemic poses a threat to public health on a global scale, and an intervention to prevent continued spread of SARS-CoV-2 virus is of the utmost importance. To assess preventive and therapeutic strategies, it is imperative to understand the pathogenesis and immune response against SARS-CoV-2. In this review, we concentrate on the protective adaptive immune response elicited by this novel coronavirus as well as requirements for a successful vaccine inducing optimal protection.

## Introduction and background

The severe acute respiratory syndrome coronavirus 2 (SARS-CoV-2) pandemic has hit the human species at a planetary scale not seen since the 1918 influenza pandemic. This new pandemic has put the global economy to a halt, and it is urgent to restore the economy in the safest possible way. There are discussions among experts of varied disciplines about “certificates of immunity” that would be conferred to convalescent patients who developed protective immunity due to the infection [1]. People who would be considered immune to the virus, and thus, will not transmit it, would be free to return to normal routines. The epidemiology, pathophysiology, clinical presentation, and diagnosis of SARS-CoV-2 have been reviewed elsewhere [2]. One pertinent but unanswered critical question is what constitutes protective immunity for those who are exposed to the SARS-CoV-2 virus. Clinical and healthcare policies in response to this pandemic rely heavily on the answers to this issue. To begin to answer this question, it is important to understand the kinetics, duration, and correlation of immunological parameters with protective immunity to other viruses, in particular to coronaviruses, and compare it with the data emerging from SARS-CoV-2. There is also an urgent need to develop successful vaccines to achieve herd immunity. Researchers have developed very successful veterinary and human vaccines in the past. Nevertheless, there were some failures with experimental viral vaccines that induced harm in people who were exposed to the virus after vaccination, a risk that should be avoided in the race to an efficacious SARS-CoV-2 vaccine [3-5]. Therefore, the understanding of what constitutes protective immunity against SARS-CoV-2 also has important implications in the development of effective and safe vaccines.

## Review

Kinetics of antibody production in patients infected with SARS-CoV-2

Using an enzyme-linked immunosorbent assay (ELISA) sensitized with the receptor-binding domain (RBD) of the Spike (S) protein, researchers were able to detect anti-RBD IgM antibodies in 73% of the patients and anti-RBD IgGs in 54% of the patients at day 14 after disease onset in 173 COVID-positive patients. Most patients seroconverted for IgM (94.3%) and IgG (79.8%) between days 15 and 39 after disease onset [6]. SARS-CoV-2 induces a more severe disease in old adults, and the induction of neutralizing antibodies early in the infection may play a role in protection. Interestingly, total antibody titer strongly correlated with disease severity in this cohort of patients. Okba et al. developed an ELISA sensitized with trimers of the whole ectodomain of the S protein, S1 (N-terminal ectodomain containing the RBD), S1A (N-terminal ectodomain not containing the RBD), the RBD, and SARS-CoV nucleoprotein (N) protein [7]. In serum samples from three patients with PCR-confirmed SARS-CoV-2 infection (one with severe disease and the other two with mild disease), seroconversion to IgG and IgA was detected between 13 and 21 days after disease onset. The most clinically affected patient seroconverted at day 13 with higher optical density (OD) and the other two at around 20 days with lesser OD by ELISA. Seroconversion was detected in all plasmas from 31 other patients at two weeks after disease onset. All ELISAs correlated strongly with 50% plaque reduction neutralization testing [7]. Thus, the presence of antibodies by ELISA (including anti-N antibodies) was correlated with the titer of neutralizing antibodies. In 14 convalescent SARS-CoV-2 patients, eight of whom were immediately post-discharge and six at two weeks post-discharge, it was shown that all but one had high titer of neutralizing antibody and the ELISA titer of anti-RBD IgG antibodies was correlated with the neutralizing antibody titers [8].

Duration of antibodies in convalescent patients infected with coronaviruses related with SARS-CoV-2

Understanding the duration of SARS-CoV-2 antibody responses will be key in addressing the question of sustained protection from reinfection. Of all coronaviruses that can infect humans, SARS-CoV-2 has the closest genetic resemblance to SARS-CoV [9]. A study of 74 convalescent SARS-CoV patients who were tested from the 2002 outbreak showed the presence of antibodies in all patients’ plasma by ELISA [10]. The antigen for this ELISA was a lysate from infected cells. IgG antibodies persisted at a detectable level for up to 720 days post-infection above the cut-off. Importantly, neutralizing antibodies persisted in most patients until day 720, though the titer decreased 100 times compared to antibodies elicited at the time of the infection. In another study, it was found that 56% of convalescent patients were positive at three years post-infection and the level of antibodies significantly decreased by the third-year post-infection [11]. These results suggest that the titer of neutralizing antibodies at two weeks post-infection is correlated with duration of immunity, and these antibodies are present up to two years after infection with SARS-CoV. As discussed above, there is also a positive correlation with severity of COVID-19 and titer of antibodies detected by ELISA against the S and N proteins [6,7]. Thus, a patient who suffered a severe disease may carry neutralizing antibodies for a longer period of time compared with patients with mild disease.

One of the target cells for SARS-CoV and SARS-CoV-2 is monocytes. It has been postulated that SARS-CoV-2 could induce antibody-dependent enhancement (ADE) of infection and/or induce Fc receptor-mediated inflammation due to the presence of anti- S non-neutralizing or subneutralizing antibodies [12]. Therefore, a previous infection with SARS-CoV-2 inducing low level of antibodies or waning of antibodies after an infection could potentially cause exacerbation of the disease in re-infected patients.

Correlation of antibody titers with protection

The question remains of what constitutes antibody-related immunity to SARS-CoV-2, and whether the serological response to infection shown by ELISA is protective for a significant period of time. Thus far, there has been no definitive study on the titer of neutralizing antibodies necessary for protection from SARS-CoV-2. The nature of humoral and cell-mediated immune responses against other respiratory viruses could provide some insight as to what that response against SARS-CoV-2 may entail. For example, a titer of 40 hemagglutination inhibition (HAI) assay units is considered protective against infection with influenza virus of the same strain [13]. The HAI detects anti-hemagglutinin antibodies (the viral attachment protein) and thus, HAI units correlate with neutralization titer. An experimental model of passive transfer of antibodies followed by challenge with porcine arterivirus (another member of order Nidovirales) in piglets was used to quantify protection with neutralizing antibodies. A titer of 1:8 of neutralizing antibodies in blood was sufficient to block viremia but it did not block local replication in lungs and lymph nodes. A titer of 1:32 or above was necessary to induce sterilizing immunity [14]. Similar experiments could be performed with non-human primates to determine the titer of neutralizing antibodies that are protective from infection with SARS-CoV-2.

Seasonal coronaviruses, such as 229E, OC43, HKU1, and NL63, are responsible for 10%-35% of upper respiratory tract infections in humans. Volunteers infected with 100 tissue culture infection doses 50% (TCID 50) of 229E presented with statistically significant (p<0.05) mild lymphopenia [15]. The kinetics of IgA and IgG antibodies in serum and mucosal IgA was determined using ELISA plates sensitized with inactivated 229E virus. The maximum concentration of circulating coronavirus IgG antibodies was found at 12-14 days. Ten of the 15 volunteers were successfully infected and experienced a transient increase in serum-specific IgA and IgG within eight days of inoculation reaching a peak at week 12. The five volunteers who were not successfully infected had a higher baseline level of mucosal IgA antibodies and reached a peak at week 2 post-challenge. These results suggest that antibodies elicited by previous infection with 229E virus provided protection to the challenge. After week 12, the kinetics of infected and uninfected volunteers was similar. Upon re-challenge one year later, all five of the unsuccessfully infected volunteers and six of nine of the successfully infected patients were infected. All had a lower mean time of viral shedding and less severe symptoms, indicating some degree of lasting protection. However, no clear antibody titer has been correlated with protection for any coronavirus as has been shown with influenza. When data from experiments of protection with neutralizing antibodies in non-human primates are available, it will be possible to predict protection with antibodies detected by ELISA plates sensitized with SARS-CoV-2 RBD and/or whole S protein [7].

Hence, there is a need for diagnostic tests capable of determining which individuals are protected from a re-infection with SARS-CoV-2. ELISAs enable the quantification of the level of antibodies. The optical density reading of anti-RBD antibodies should be correlated with the titer of neutralizing antibodies.

Protective cellular adaptive immune response against SARS-CoV and MERS-CoV

Investigations of the cellular immune response to other related coronaviruses can provide insight into what is needed to generate protection from SARS-CoV-2. Memory CD8+ T cells were protective from lethal SARS-CoV infection in mice after immunization with peptide-loaded dendritic cells and boosting with SARS-CoV T-cell epitopes [16]. Two HLA-A2 specific T-cell epitopes found in the SARS-CoV S protein were shown to elicit a strong and likely protective CD8+ T-cell response in patients who recovered from SARS virus infection [17]. These results in a mouse model and in humans suggest that a T-cell response is important in protection against an infection with SARS-CoV.

Failure to generate a sufficient cell-mediated response may prove costly in coronavirus infections. SARS-CoV-2 can infect T-cell lines via S protein-mediated endocytosis with greater efficiency than SARS-CoV virus [18]. While Middle East respiratory syndrome coronavirus (MERS-CoV) in particular has been shown to induce T-cell-specific apoptosis, the same was not found for SARS-CoV and has yet to be shown for SARS-CoV-2 [19]. Nevertheless, prolonged CD4+ and CD8+ lymphopenia was observed in patients with SARS-CoV and associated with more severe illness and death [20]. Profound lymphopenia was also associated with and observed until death in SARS-CoV-2 non-survivors, whereas lymphopenia improved in survivors [21]. Lymphopenia in SARS-CoV-2 consists of depletion of both CD4+ and CD8+ T cells with no significant alteration in CD4+/CD8+ ratio [22]. These authors showed that severity of lymphopenia in COVID-19 patients positively correlates with severity of disease. Patients responsive to treatment showed an increase of CD8+ T cells after a week, whereas there was not an increase of CD8+ T cells in non-responsive patients. Thus, level of CD8+ T cells can be used as a predictor of clinical outcome. Increase of CD8+ T cells is typical in infections with respiratory viruses, and the reason for the depression of CD8+ T cells in infections with novel coronaviruses is not fully understood. SARS-CoV-2 shares several non-structural proteins with SARS-CoV, which induce a strong decrease of the type I interferon (IFN) response in infected cells [23]. Secretion of type I IFNs dramatically increases CD8+ T cells response against viruses [24]. Thus, the down-regulation of type I IFNs by different non-structural proteins of SARS-CoV-2 could help to explain the absence of a strong CD8+ T-cell response in patients with COVID-19. The use of type I IFNs is a promising treatment against infection with SARS-CoV-2 [25]. Besides the anti-viral effect of type I IFNs, the use of these IFNs early in the infection could also help to potentiate the CD8+ T cells. This action is mediated by increased expression of HLA molecules resulting in an increase in antigen presentation by dendritic cells.

Of note, the cellular immune protection offered by infection also has the potential to be more persistent than antibody-mediated responses. Both CD4+ and CD8+ SARS-CoV-specific memory T cells have been shown to persist in convalescent SARS-CoV patients at low frequency up to six years post-infection [26]. Stimulation of peripheral blood mononuclear cells (PBMCs) from a convalescent patient with peptides from the Matrix (M) and N proteins of SARS-CoV was also shown to elicit a CD8+ T-cell response 11 years post-SARS-CoV infection [27]. It has recently been shown that the IFN-gamma-secreting T cells specific for SARS-CoV-2 are produced in convalescent patients and that the production of NP-specific T cells correlates with neutralizing antibody titers [8]. Since there is a high-sequence identity between proteins from other beta coronaviruses, including the NP, it is also possible that heterotypic cellular immunity may play a protective role against SARS-CoV-2 in individuals previously infected with other coronaviruses such as 229E [9]. Therefore, the different presentations of the disease in patients infected with SARS-CoV-2 could be related to some extent with CD8+ T-cell memory to other coronaviruses. For instance, a proteomic comparative analysis of the four common coronaviruses and SARS-CoV-2 demonstrated that HLA-B* 15:03 shows the greatest capacity to present peptides shared between the common coronaviruses and SARS-CoV-2 [28]. Thus, further investigation into the profile of T cells affected and the persistence of this response in patients with SARS-CoV-2 is warranted.

Cellular immunity is important in protection against SARS-CoV-2 and related viruses. Thus, in addition to detection of antibodies, it is important to develop commercially available gamma-interferon release assays that will be able to correlate the level of cellular immunity. These assays could be used to predict clinical outcome in patients with COVID-19 and protection from SARS-CoV-2 in the general population.

Anti-SARS-CoV-2 RNA and replication-deficient adenovirus vaccines

The titer of neutralizing antibodies that correlates with protection against infection with SARS-CoV-2 is not known. Therefore, there are no parameters to precisely determine the potency of anti-SARS-CoV-2 vaccines. In addition, vaccine-induced or natural antigenic drift could create another problem with SARS-CoV-2: the presence of sub-neutralizing antibodies that may induce antibody-dependent enhancement of infectivity [12]. Therefore, it is of the utmost importance to develop vaccines inducing a high titer of neutralizing antibodies to decrease the possibility of vaccine-induced ADE, as well as strong cellular immunity.

Several non-replicative vaccines against SARS-CoV-2 are currently under clinical trials. Of note are replication-deficient recombinant adenoviruses encoding the SARS-CoV-2 S protein (NCT04324606, ChiCTR2000031781). These vectors infect cells, but there is not viral replication in the host. As a result of the infection, there is a strong CD4+ TH1, CD8+, and neutralizing antibody response [29]. A replication-deficient recombinant adenovirus carrying the S protein of MERS CoV has proven effective in protecting vaccinated camels from viral shedding [30]. A phase I clinical trial with this vaccine showed no relevant adverse effects and the induction of neutralizing antibodies as well as cellular immunity. The level of neutralizing antibodies decreased at one year post-vaccination, but cellular immunity lasted at a high level up to a year post-vaccination [31]. Previous results obtained with this vaccine suggest that a SARS-CoV-2 replication-deficient recombinant adenovirus vaccine could be successful in inducing protective immunity.

Several nucleic acid vaccines are also under clinical trial. Of note are RNA vaccines encoding for SARS-CoV-2 S (NCT04283461, 2020-001038-36, NCT04368728). There is plenty of experimental data, mainly with influenza virus, showing that this type of vaccine induces cellular and antibody immunity. Experiments in mice and macaques showed a strong induction of CD4+ TH1 and CD8+ T cells and neutralizing antibody response above the threshold of protection against several types of influenza viruses [32,33]. A self-amplifying mRNA (SAM) vaccine encoding the hemagglutinin gene of influenza virus induced strong neutralizing antibody response and CD4+ TH1 and CD8+ T-cell responses and conferred protection to challenge in mice [34]. Recently, two randomized, placebo-controlled, double-blind, phase I clinical trials were reported using mRNA vaccines against two influenza strains (H10N8 and H7N9) [35]. The vaccines were lipid nanoparticle-formulated mRNA vaccines given at various dosages by the intramuscular and intradermal routes. Seroconversion rates for both vaccines at the higher doses were comparable to squalene-adjuvanted H7N9 influenza vaccines and detectable HAI titers were present up to six months post-vaccination. No serious adverse effects were shown with these vaccines. Therefore, results in experimental models and in clinical trials showed that RNA vaccines are capable of producing strong cellular and antibody titers comparable to licensed influenza vaccines.

Vaccines against SARS-CoV-2 should induce a strong long-lasting level of neutralizing antibodies and cellular immunity. A strategy combining an RNA vaccine or a replication-deficient recombinant virus, followed by a booster with an RBD protein subunit vaccine, could help to induce a strong protective response against SARS-CoV-2 by potentiating both branches of the adaptive immune system. The priming vaccine doses will induce cellular and antibody responses and the RBD vaccine could further induce higher titers of neutralizing antibodies. 

## Conclusions

Cellular immunity and neutralizing antibodies are important in protection against infection with SARS-CoV-2. There is a need to develop ELISAs to be able to quantify the level of anti-RBD antibodies and its correlation with the level of neutralizing antibodies providing protection from re-infection. There is also a need to develop gamma-interferon release assays to determine the level of cellular immunity against SARS-CoV-2 correlating with protection. Likewise, anti-SARS-CoV-2 vaccines must induce strong cellular immunity and a high titer of neutralizing antibodies to fully protect vaccinated individuals. 
